# Machine learning reveals microbial interactions driving plastic degradation across plastisphere environments

**DOI:** 10.3389/fmicb.2025.1691658

**Published:** 2026-01-23

**Authors:** Akib Al Mahir, Arjun Sathyan Kulathuvayal, Yunjian Lei, Qijun Zhang, Luguang Wang, Yanqing Su, Liyuan Hou

**Affiliations:** 1 Utah Water Research Laboratory, Logan, UT, United States; 2 Department of Civil and Environmental Engineering, Utah State University, Logan, UT, United States; 3 School of Aerospace and Mechanical Engineering, University of Oklahoma, Norman, OK, United States; 4 Computer Sciences Department, University of Wisconsin-Madison, Madison, WI, United States; 5 Department of Bacteriology, University of Wisconsin-Madison, Madison, WI, United States; 6 Department of Biological Engineering, Utah State University, Logan, UT, United States

**Keywords:** plastic degradation, predictive modeling, microbial analysis, machine learning, microplastic biofilm

## Abstract

Microplastic pollution fosters the development of distinct microbial biofilm communities, termed the plastisphere, that vary across environmental contexts. Here, we used 16S rRNA gene sequencing combined with machine learning (ML) approaches to explore plastisphere microbial diversity and the interactions between potential plastic-degrading bacteria (PDBs) and non-plastic-degrading bacteria (NDBs) across ocean, surface water, and wastewater habitats. Our findings reveal that wastewater plastispheres harbor the most diverse and compositionally even microbial communities, likely driven by complex nutrient loads, pollutant inputs, and high microbial seeding potential. Genus-level analysis of potential PDBs indicated habitat-specific taxa, including *Pseudomonas*, *Acinetobacter*, and *Aquabacterium* in wastewater, *Flavobacterium* and *Alteromonas* in ocean, and *Psychrobacter* and *Novosphingobium* in surface waters. Network analyses using Pearson’s correlation and Random Forest modeling uncovered consistent co-occurrence patterns between potential PDBs and diverse NDB taxa such as *Clostridium_sensu_stricto_5*, *Lachnospiraceae_UCG-001*, and *Cloacibacterium*, suggesting potential facilitative interactions, including redox modulation, nutrient exchange, and biofilm support. ML tools proved effective in identifying key taxa and potential ecological interactions, but their application remains limited by taxonomic resolution, lack of functional validation, and insufficient integration of environmental metadata. These findings underscore the ecological complexity of plastisphere communities and the need for community-level approaches in plastic biodegradation research.

## 1 Introduction

Plastic production has grown rapidly due to its unique properties, increasing 20-fold since 1964 and reaching 460 million tons worldwide in 2024 (Urbanek et al., 2018). Approximately 91% of plastic waste globally remains unrecycled, with the majority accumulating in landfills or the ocean (Hou et al., 2021). This widespread and persistent plastic pollution threatens ecosystems, biodiversity, and human well-being (Hou et al., 2021). Through various chemical, physical, and biological processes, plastics from landfills, oceans, and other sources are weathered into smaller fragments known as micro- or nano-plastics (Hou et al., 2021). Microplastics, defined as plastic particles less than 5 mm in size, are of particular concern due to their potential to accumulate toxic substances and be ingested by organisms (Liao et al., 2023; Shatara et al., 2025). Once released into natural environments, plastics are quickly colonized by diverse microbial communities, forming the *plastisphere*, a unique ecological niche on plastic surfaces. The plastisphere not only contributes to plastic degradation but also acts as a potential vector for pathogens, invasive species, and antibiotic resistance genes. Studying the plastisphere is essential for understanding the environmental fate of plastics, their ecological impacts, and for advancing microbe-based solutions for plastic biodegradation (Dey et al., 2022).

A total of 824 microbial species have been reported to possess plastic-degrading capabilities, along with 230 proteins identified as being involved in plastic breakdown. Among these species, approximately half are bacteria, and the other half are fungi (Gambarini et al., 2022). PDBs degrade synthetic polymers into simpler compounds through specialized enzymatic activity. These bacteria colonize plastic surfaces in terrestrial and aquatic environments, secreting extracellular enzymes such as PETase, cutinase, laccase, and esterase, which initiate the depolymerization of plastics into smaller oligomers or monomers that then undergo further biodegradation and mineralization (Hou and Majumder, 2021). However, many enzymes involved in plastic degradation remain unidentified or poorly characterized. Only members of specific genera, such as *Ideonella*, *Pseudomonas*, *Acinetobacter*, and *Bacillus*, etc., have well-defined enzymatic pathways (Narancic and O’Connor, 2017; Patrício Silva, 2021). For example, *Ideonella sakaiensis* degrades polyethylene terephthalate (PET) into mono(2-hydroxyethyl) terephthalate (MHET) and terephthalic acid (TPA), which are further metabolized via terephthalic acid catabolic pathways (Yoshida et al., 2016; Sevilla et al., 2023). *Pseudomonas* species are active in degrading polyethylene (PE) and polyurethane (PU) through extracellular hydrolases and oxidoreductases (Wilkes et al., 2017). Members of *Bacillus*, including *B. subtilis* and *B. licheniformis*, can degrade PU through oxidoreductase-mediated pathways (Gui et al., 2023). Thus, studying PDBs within the plastisphere represents a promising strategy for understanding and harnessing the potential of microbial biodegradation.

Plastisphere communities vary widely across marine, freshwater, and wastewater systems, shaped by abiotic factors such as salinity, pH, temperature, and nutrient availability, as well as biotic interactions (Amaral-Zettler et al., 2020; Oberbeckmann and Labrenz, 2020). Marine plastisphere is enriched with hydrocarbon-degrading bacteria, such as *Alcanivorax* and *Alteromonas* (Zettler et al., 2013), while freshwater plastispheres are dominated by *Flavobacteriaceae* and *Sphingomonadaceae* (Bocci et al., 2024). In wastewater-associated plastisphere communities, *Pseudomonas* is frequently accompanied by other taxa such as *Aeromonas*, *Bacillus*, and *Sphingomonas* (Kelly et al., 2021; Witsø et al., 2024). These differences highlight the roles of environmental filtering and microbial competition in shaping PDB profiles. Despite increasing interest in plastisphere ecology, systematic comparisons of plastisphere composition and PDB profiles across diverse aquatic environments remain scarce.

PDBs and non-plastic-degrading bacteria (NDBs) engage in complex ecological interactions that shape both the efficiency of plastic biodegradation and the structural dynamics of the plastisphere. Such interactions, including cross-feeding, environmental stabilization, enzymatic cooperation, and biofilm facilitation, may collectively enhance plastic degradation performance. In cross-feeding, PDBs depolymerize plastics into intermediates, such as MHET and terephthalic acid from PET by *Pseudomonas* spp., which can be metabolized by microbes lacking depolymerization enzymes (Yoshida et al., 2016; Danso et al., 2019). NDBs also stabilize microenvironments by regulating pH, oxygen, and redox conditions, and by producing cofactors or vitamins essential for PDB activity. For example, facultative anaerobes like *Escherichia-Shigella* or *Cloacibacterium* consume excess oxygen or acidic byproducts, protecting enzymes in PDBs such as *Acinetobacter* or *Pseudonocardia* from denaturation (Wright et al., 2020; Gao and Sun, 2021). Enzymatic cooperation between PDBs and NDBs, as observed with *Rhodobacteraceae* co-occurring with *Alcanivorax* and *Pseudomonas* on PE and polyhydroxyalkanoate films, further enhances polymer breakdown (Pinnell and Turner, 2019). Biofilm facilitation by certain NDBs, including *Flavobacterium* and *Sphingomonadaceae*, provides the structural stability through extracellular polymeric substances, enabling colonization and localized enzymatic activity of PDBs (Oberbeckmann et al., 2016). However, not all interactions between PDBs and NDBs are beneficial, as some NDBs outcompete PDBs for nutrients or surface attachment, thereby reducing plastic degradation efficiency (Urbanek et al., 2018; Danso et al., 2019). Efforts are required to identify and evaluate the interactions between PDBs and NDBs, as many PDBs have been reported but their enzymatic pathways remain largely uncharacterized, calling for innovative tools.

Recent advances in artificial intelligence (AI) and microbial analysis offer powerful tools for studying plastic degradation across diverse environments. AI modeling techniques, including machine learning (ML) and deep learning, can analyze large datasets and reveal complex patterns beyond traditional statistical methods (Kourou et al., 2015). Microbial analysis techniques, including 16S rRNA sequencing and metagenomics, enable comprehensive profiling of microbial communities and their functional potential (Shakya et al., 2020). Integrating AI modeling with microbial analysis allows the development of predictive models to identify potential PDB and their microbial partners. AI have also shown promise in modeling and predicting complex degradation processes. Hemalatha et al. (2021) applied ML classifiers, such as Decision Trees, Random Forests, and Support Vector Machines, achieving up to 99.1% accuracy in predicting plastic-degrading microbes. Malwe et al. (2025) introduced XenoBug, a ML-based tool to identify both known and potential plastic-degrading enzymes, including monooxygenases and hydrolases from *Pseudomonas*, *Bacillus*, and *Streptomyces* (Malwe et al., 2025). These advancements highlight the potential of AI to accelerate the discovery of potential PDBs and their functional enzymes, thereby guiding bioremediation strategies. By integrating AI modeling with microbial analysis, potential PDB–NDB interactions can be elucidated, gain deeper insights into plastic degradation dynamics, and design targeted interventions for enhanced efficiency.

Therefore, this research aims to advance the understanding of plastic degradation across diverse environments and plastisphere profile. We address this need by systematically analyzing plastisphere biofilms across ocean, surface water, and wastewater environments using unified data processing and analytical pipeline applied to publicly available 16S rRNA sequencing datasets. The objectives of this study are: (1) to comprehensively profile the microbial communities within the plastisphere across diverse habitats; (2) to identify core potential PDB and their associated NDB partners in various environmental contexts; and (3) to apply ML models capable of predicting potential PDB across environments. As both 16S rRNA taxonomic resolution and many PlasticDB annotations are primarily limited to the genus level, we define potential PDBs as genera containing known degraders. Although this may overestimate true PDBs, our ML results reflect abundance patterns, not enzyme activity, revealing community-level associations. These insights will support the development of engineered microbial systems for efficient plastic removal and remediation. We hypothesize that (i) different environments harbor distinct potential PDBs with unique degradation capabilities, and (ii) potential PDBs are associated with specific NDBs that facilitate plastic degradation through ecological interactions.

## 2 Methods and materials

### 2.1 Collection and preprocessing of 16S rRNA sequences from NCBI SRA

Data collection for this research involved the acquisition of 16S rRNA sequencing data from the National Center for Biotechnology Information (NCBI) Sequence Read Archive (SRA) and curated entries from Plastic Degrader Database (PlasticDB). Additionally, a comprehensive literature survey was conducted to identify relevant studies focusing on microbial biofilms on microplastics across marine, wastewater, and surface water environments (Table 1). To ensure data quality and comparability, three representative studies were selected from each environment (ocean, wastewater, and surface water) based on the availability of raw 16S rRNA sequencing data in NCBI SRA, comprehensive metadata, and clear relevance to microplastic-associated biofilms or plastisphere. These studies were prioritized for their use of well-defined plastic types, controlled experimental designs, and consistent sequencing methods, which facilitate a unified data processing pipeline. Selecting a limited number of high-quality datasets per environment allows for robust comparative analysis while maintaining computational feasibility. The corresponding FASTQ files were downloaded from the NCBI SRA using the *fastq-dump* tool (NCBI SRA Toolkit) through a custom Bash script (.sh) to automate retrieval. The downloaded files were subsequently verified, reformatted, and organized for downstream analyses, including quality control and operational taxonomic unit (OTU) construction. Metadata associated with each dataset (e.g., sampling location, polymer type, and environmental parameters) was initially intended for collection (Table 1); however, due to incomplete records and lack of consistent connections across samples, it was not utilized in the subsequent analyses. In addition, 16S rRNA sequencing data from control or background samples not related to plastisphere communities, as indicated in the SRA descriptions, were excluded from further analysis.

**Table 1:** Overview of microplastic exposure studies and associated sample metadata from NCBI SRA.

Environment	Number of samples	Experimental methods and plastic types	Bioproject ID	NCBI SRA link
Ocean	75 (includes surface seawater, blank, and glass controls)	*In situ* incubations of plastics at the sea surface. Plastic types: PP, HDPE, LDPE, PVC-DINP, PVC-DEHP (Pinto et al., 2019)	PRJNA515271	https://www.ncbi.nlm.nih.gov/sra/?term=PRJNA515271
Ocean	48	Clear PET bottles (500 mL) deployed at each location by SCUBA divers (6 replicates/location; 24 total). Plastic type: PET (Harvey et al., 2020)	PRJNA600662	https://www.ncbi.nlm.nih.gov/sra/?term=PRJNA600662
Ocean	154 (includes sterile water, wood-associated, river water, rinsing water, treated wastewater communities, and *Escherichia* DNA)	HDPE and PS pellets (3 mm diameter) incubated for 14 days at 5 estuarine and marine stations. Plastic types: HDPE, PS (Oberbeckmann et al., 2018)	PRJNA338729	https://www.ncbi.nlm.nih.gov/sra/?term=PRJNA338729
Wastewater	58	Seven polymer types tested: biodegradable PLA, PHB, PCL; non-biodegradable PET, POM, PS, and LDPE (Martínez-Campos et al., 2021)	PRJNA543601	https://www.ncbi.nlm.nih.gov/sra/?term=PRJNA543601
Wastewater	41 (includes free water surface constructed wetland and municipal sewage)	HDPE, PVC, PET, and PS infusers attached to chains and mesh bags; deployed along wastewater treatment processes at three sites (Bydalek et al., 2023)	PRJNA940639	https://www.ncbi.nlm.nih.gov/sra/?term=PRJNA940639
Wastewater	28	Semi-continuous chemostat cultures seeded with 80% Lake Maggiore water and 20% WWTP effluent. Plastic types: PET, tire wear particles (Sathicq et al., 2022)	PRJNA704794	https://www.ncbi.nlm.nih.gov/sra/?term=PRJNA704794
Surface Water	30	PE fibers tied to wooden poles at fixed seawater depth. Plastic type: HDPE (Jin et al., 2020)	PRJNA602404	https://www.ncbi.nlm.nih.gov/sra/?term=PRJNA602404
Surface Water	48 (includes 18S amplicon data)	~3 mm plastic particles exposed in Truppach freshwater stream, ~500 m downstream from a WWTP. Plastic types: PE, PP, PS, PVC (Weig et al., 2021)	PRJNA680706	https://www.ncbi.nlm.nih.gov/sra/?term=PRJNA680706
Surface Water	45	MPs enclosed in metallic cages deployed for 11 days (25/07/2017–04/08/2017) at 20 cm lake depth. Plastic types: PHB, LDPE, HDPE (González-Pleiter et al., 2021)	PRJNA722376	https://www.ncbi.nlm.nih.gov/sra/?term=PRJNA722376

PP, polypropylene; HDPE, high-density polyethylene; LDPE, low-density polyethylene; PVC, polyvinyl chloride; DINP, diisononyl phthalate; DEHP, di(2-ethylhexyl) phthalate; PET, polyethylene terephthalate; PS, polystyrene; PLA, polylactic acid; PHB, poly(3-hydroxybutyrate); PCL, polycaprolactone; POM, polyoxymethylene.

### 2.2 OTUs generation and taxonomy annotation

**Figure 1:**
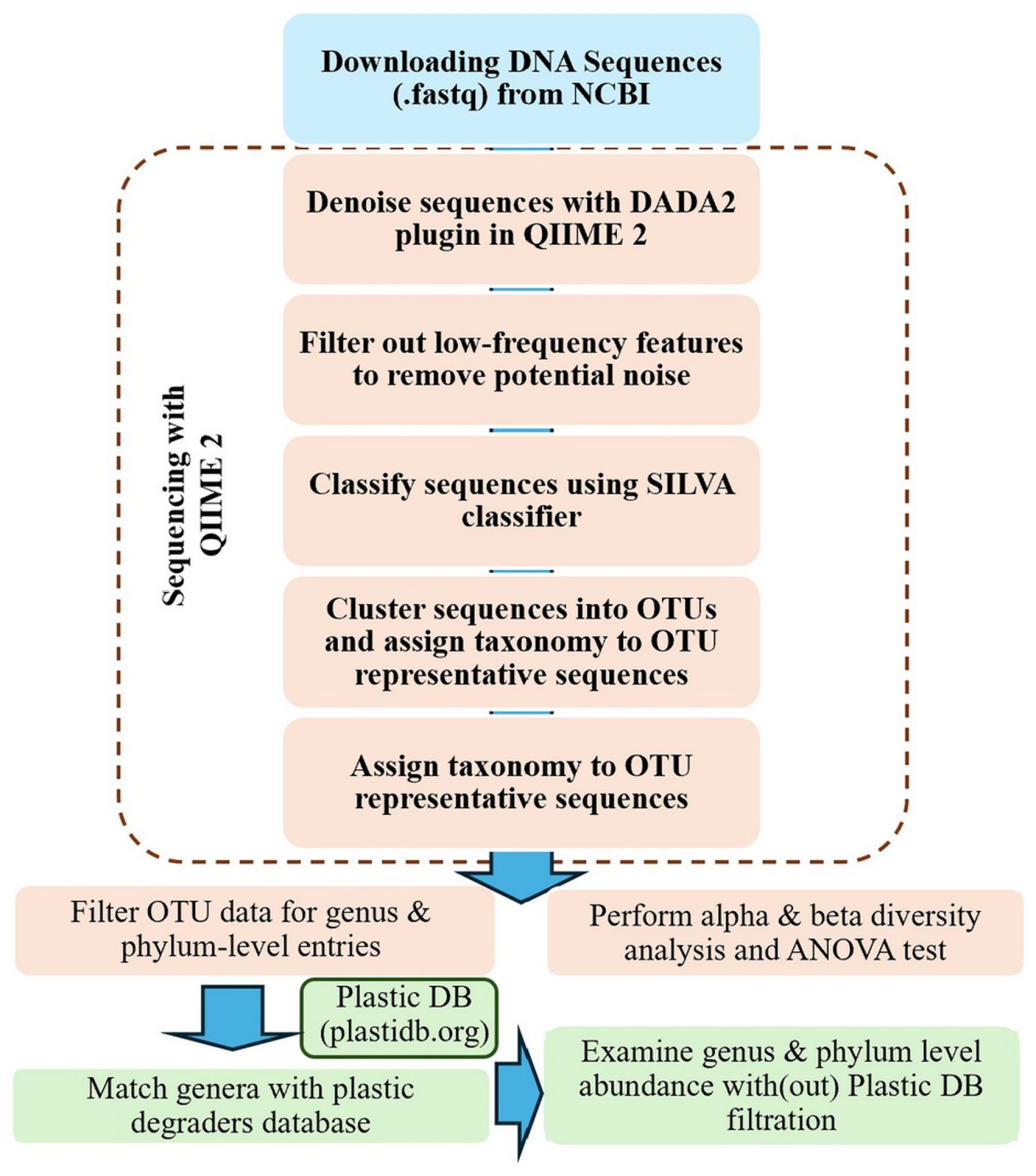
A systematic workflow for processing FASTQ data from published NCBI datasets used in this study.

Operational Taxonomic Units (OTUs) construction and taxonomic classification were performed using the QIIME2 (v2023.2) pipeline, following a systematic workflow (Figure 1; Bolyen et al., 2019; Hou et al., 2021). Raw paired-end FASTQ reads from NCBI SRA were first demultiplexed and quality-checked using *qiime demux* and *qiime quality-filter q-score*. Reads were then denoised using the DADA2 plugin to remove chimeras, correct sequencing errors, and obtain high-quality representative sequences. To reduce noise, low-abundance features such as singletons were filtered out based on abundance thresholds. The high-quality sequences were then clustered into OTUs at a 97% similarity threshold using the qiime vsearch cluster-features-de-novo plugin, while identical sequences were dereplicated to avoid redundancy. Representative OTU sequences were aligned with qiime alignment mafft, and a phylogenetic tree was generated using qiime phylogeny fasttree to support diversity analyses. Taxonomic classification of OTU representative sequences was performed using a pre-trained SILVA 138 classifier (*qiime feature-classifier classify-sklearn*), assigning taxonomy from phylum to genus levels. The OTU table was reformatted with samples as rows and taxonomic classes as columns, and habitat metadata (e.g., environment type and plastic type) was integrated for downstream alpha- and beta-diversity analyses. Finally, the curated OTU dataset was used for AI-based modeling to identify PDBs and their associated NDBs across different environments. Additionally, a substantial number of sequences could not be assigned to known taxonomic groups and were therefore omitted from further analysis. The surface water dataset includes only 72 samples, as low-quality sequences were removed due to insufficient quality scores, rendering them unsuitable for reliable taxonomic classification.

### 2.3 Bacterial community analysis of microbiome diversity

The alpha diversity metrics (Shannon, Simpson, Simpson, Chao1, Species Richness) were calculated and compared across plastisphere microbial communities in ocean, surface water, wastewater environments. Statistical significance among these environments was evaluated using ANOVA tests in R, which revealed significant variations in microbial diversity. Box plots and jitter plots were generated to visualize the diversity indices by environment, annotated with observation counts. The beta diversity analysis employed Bray–Curtis dissimilarity, followed by Principal Coordinates Analysis (PCoA) in R, to assess community composition differences among environments. Beta diversity differences among plastisphere communities from ocean, wastewater, and surface water were assessed using Permutational Multivariate Analysis of Variance (PERMANOVA) with 999 permutations using the *adonis* function in the vegan R package (2.6–10).

### 2.4 Taxonomic identification of potential PDBs using PlasticDB

To identify potential plastic-degrading microorganisms (PDMs) in the plastisphere, the OTU table obtained from 16S rRNA gene sequencing was compared against the PlasticDB database.^1^ PlasticDB is a curated repository of microorganisms, enzymes, and genes experimentally validated to be involved in plastic biodegradation. The PlasticDB dataset was downloaded, and genus names were extracted from the “microorganism” column. This list represents all genera, phyla, and classes reported to harbor species capable of plastic degradation across different polymer types. The initial OTU data were processed and filtered to retain only genus-level entries (marked by “g__”), while entries lacking genus-level classification were excluded. Genus names were extracted and standardized to remove ambiguous or unclassified labels. To examine higher-level taxonomic patterns, phylum information was extracted from the PlasticDB lineage column using a regular expression to parse text between “phylum” and “class.” The OTU table was similarly filtered to retain entries with phylum-level classification (taxa containing the “p__” prefix). Only OTU entries belonging to phyla reported in PlasticDB were retained for subsequent diversity and comparative analyses. It should be noted that our analysis does not provide species-level identification. Instead, we identify genera or classes that contain species previously reported to degrade plastics as both 16S resolution and PlasticDB records are largely restricted to the genus level. This approach may overestimate the occurrence of plastic-degrading microorganisms, since not all members of a given genus necessarily possess this capability. Accordingly, our estimates should be interpreted as upper-bound values appropriate for hypothesis generation. All subsequent analyses and ML models were based on relative abundance profiles, not enzymatic activity, ensuring that observed relationships reflect community-level ecological associations rather than assumed degradation functions. Nonetheless, it offers a valuable overview of the potential distribution and relative abundance of plastic-degrading taxa across plastisphere communities.

### 2.5 Statistical and machine learning analysis of potential PDB and NDB relationships

Building upon the taxonomic profiling and PlasticDB-based identification of potential PDB, we further explored the relationships between potential PDB and NDB using statistical and ML approaches. The analysis was to (1) evaluate potential relationships between potential PDB and NDB and (2) identify key NDB features that could influence potential PDB abundance (Supplementary Figure S1), thereby offering deeper insights into microbial community dynamics across different environments. Linear associations were first examined using Pearson’s correlation analysis to quantify the strength and direction of linear relationships between potential PDB and NDB. The Pearson correlation coefficient, r(f1,f2) , between feature f1 and feature f2 was calculated using Equation 1:

$r(f1,f2)=\frac{\sum (f1_{i}-\bar{f}1)(f2_{i}-\bar{f}2)}{\sqrt{\sum {\left(f1_{i}-\bar{f}1\right)}^{2}\sum {\left(f2_{i}-\bar{f}2\right)}^{2}}}$ (1)

Here, $f1_{i}$ and $f2_{i}$ are the feature values (normalized bacterial abundances, expressed as percentages, in this study), and $\bar{f}1$ and $\bar{f}2$ are the means of the data sets $f1_{i}$ and $f2_{i}$ , respectively. ‘i’ is the index that runs through the data points.

To capture potential non-linear relationships, each potential PDB genera was trained using NDB dataset with the Random Forest algorithm from the Scikit-learn Python library (Pedregosa et al., 2011). Each PDB genus was treated as the target variable, and all NDB genera served as predictive features (Supplementary Figure S1). The NDB dataset contained normalized bacterial abundance values. To reduce redundancy and prevent model overfitting, highly correlated NDB features (|*r*| > 0.95) were filtered, retaining the one with higher Mean Absolute Deviation (MAD) to preserve informative variability. This iterative process minimized multicollinearity in the high-dimensional dataset. For each target potential PDB genus, an iterative training procedure was performed by progressively including NDB features in increasing order of MAD values. Hyperparameter tuning was conducted using *GridSearch()* function with an 80:20 train-test split. Feature importance was then used to identify non-linear associations between potential PDB and NDB. This evaluation was performed for models achieving an *R*^2^ score above 0.50, and the top 15 NDB features were retained from each high-performing training model.

Normalized bacterial abundances (calculated as the percentage of OTU counts of each genus relative to the total OTU counts for all genera in each sample) from the three studied water bodies, including ocean, wastewater, and surface water, were further classified using the *KNeighborsClassifier()* function. We also evaluated a Random Forest classifier; however, K-Nearest Neighbors achieved superior classification performance and is commonly applied in similar ecological datasets (Yang et al., 2006). Principal Component Analysis (PCA) was applied to using normalized genus-level bacterial abundances to reduce the dimensionality of the feature space for potential PDB and NDB. To minimize confounding from ecological gradients such as salinity, PDB–NDB analyses were also performed separately for each environment (ocean, surface water, and wastewater), using environment-specific Pearson correlation and Random Forest models. The scripts used for the analyses are available in the GitHub repository at https://github.com/skvarjun/ML_PDmicrobes.

## 3 Results

### 3.1 Microbial diversity across habitats

**Figure 2:**
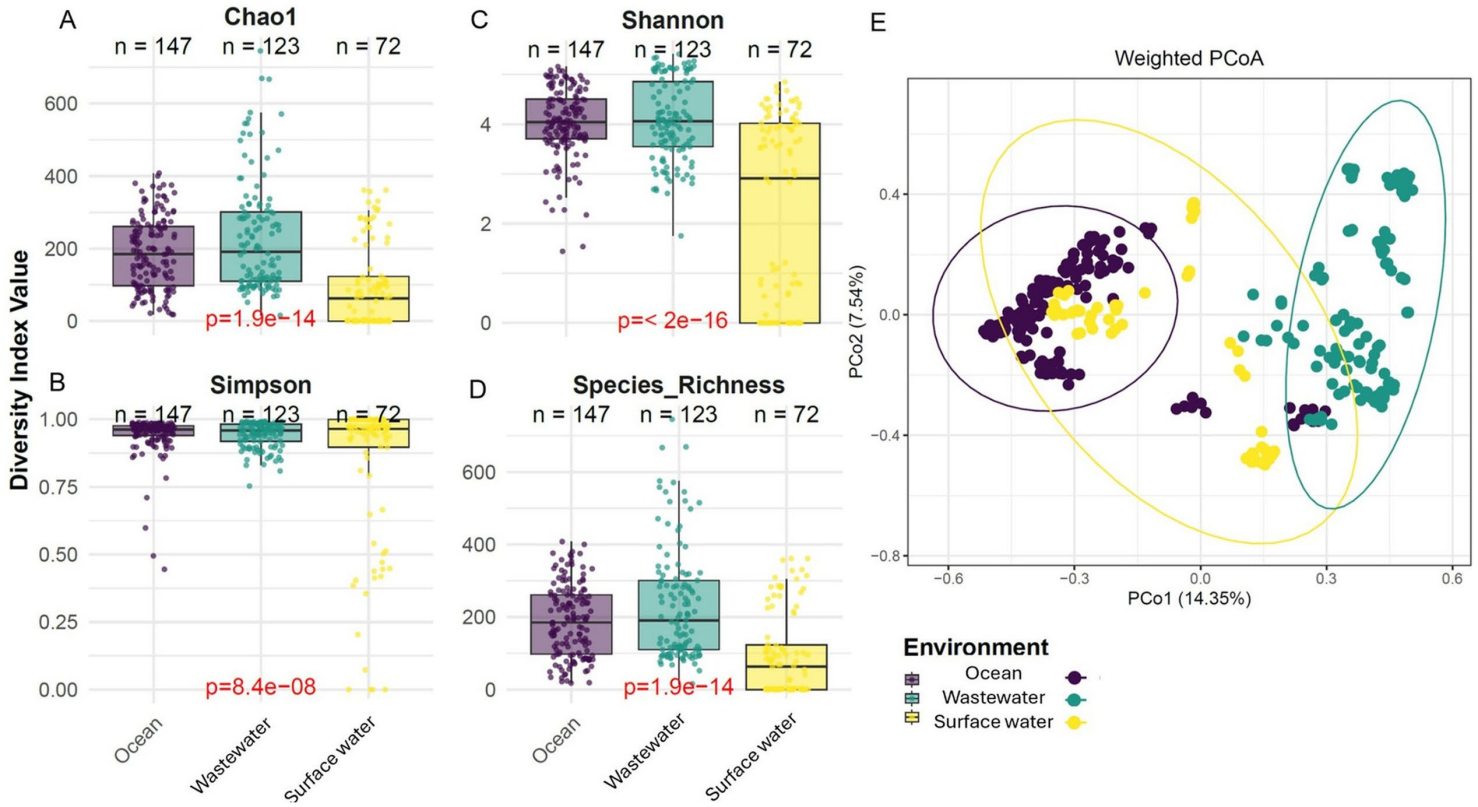
Plastisphere alpha and beta diversity in environmental samples across different habitats (ocean, wastewater, and surface water). Alpha diversity metrics, including Chao1 **(A)**, Shannon **(B)**, Simpson **(C)**, and species richness **(D)**, reveal significant differences in microbial diversity among the habitats. Boxplots display the median, interquartile range, and individual sample points for each group. **(E)** PCoA based on unweighted UniFrac distances. Each point represents a sample, and the ellipses indicate 95% confidence intervals for group centroids.

Alpha diversity analysis including Chao1, Shannon, Simpson, and Species Richness revealed significant variability in plastisphere communities among the three water bodies (Figures 2A–D). Plastisphere in wastewater exhibited the highest Chao1 (median 201) and species richness (median 205), indicating a more taxonomically diverse community compared to ocean (Chao1: 185; species richness: 190) and surface water (Chao1: 60; species richness: 65) (*p* < 0.001). For Shannon diversity, plastisphere in both ocean (median 4) and wastewater (median 4.05) showed similarly high values, suggesting balanced community evenness, while plastisphere in surface water had a lower Shannon index (2.8) (*p* < 0.001), reflecting less evenness. Simpson index values were consistently high (> 0.9) across all habitats, but plastisphere in surface water had a slightly higher median (0.94) (*p* < 0.001), indicating more dominant species coexisting with a diverse community. Across the three habitats, α-diversity differed significantly (all *p* < 0.001) with moderate effect sizes (η^2^ = 0.26, 95% CI 0.18–0.34; n(ocean) = 147, n(wastewater) = 123, n(surface) = 72). Overall, wastewater harbored the most diverse and even plastisphere community structure, while surface water exhibited the lowest plastisphere diversity across all indices. While only three studies per habitat were included, the large number of samples (>100 per habitat) encompassing diverse locations and plastic types provides sufficient coverage for robust ecological and statistical analyses. Future expansion of the dataset will allow cross-validation of these findings and further confirm the consistency of observed plastisphere diversity trends.

PCoA based on weighted and unweighted UniFrac distances revealed clear separation of plastisphere microbial communities among ocean, wastewater, and surface water (Figure 2E and Supplementary Figure S1). In the weighted PCoA plot, which accounts for relative abundances of bacterial genera, plastisphere in wastewater and ocean samples clustered distinctly (PERMANOVA *R*^2^ = 0.23, 95% CI 0.17–0.29, *p* < 0.001), while plastisphere in surface water samples were more dispersed but partially overlapped with both, indicating variations in dominant taxa among habitats (Figure 2E). The unweighted PCoA plot, which emphasizes the presence or absence of taxa, showed a similar clustering pattern with wastewater and ocean forming distinct groups and surface water positioned between them (Supplementary Figure S2), reflecting unique community compositions with some shared taxa. Together, these analyses highlight the strong influence of habitat on plastisphere microbial community structure.

### 3.2 Potential PDB profiles across habitats

**Figure 3:**
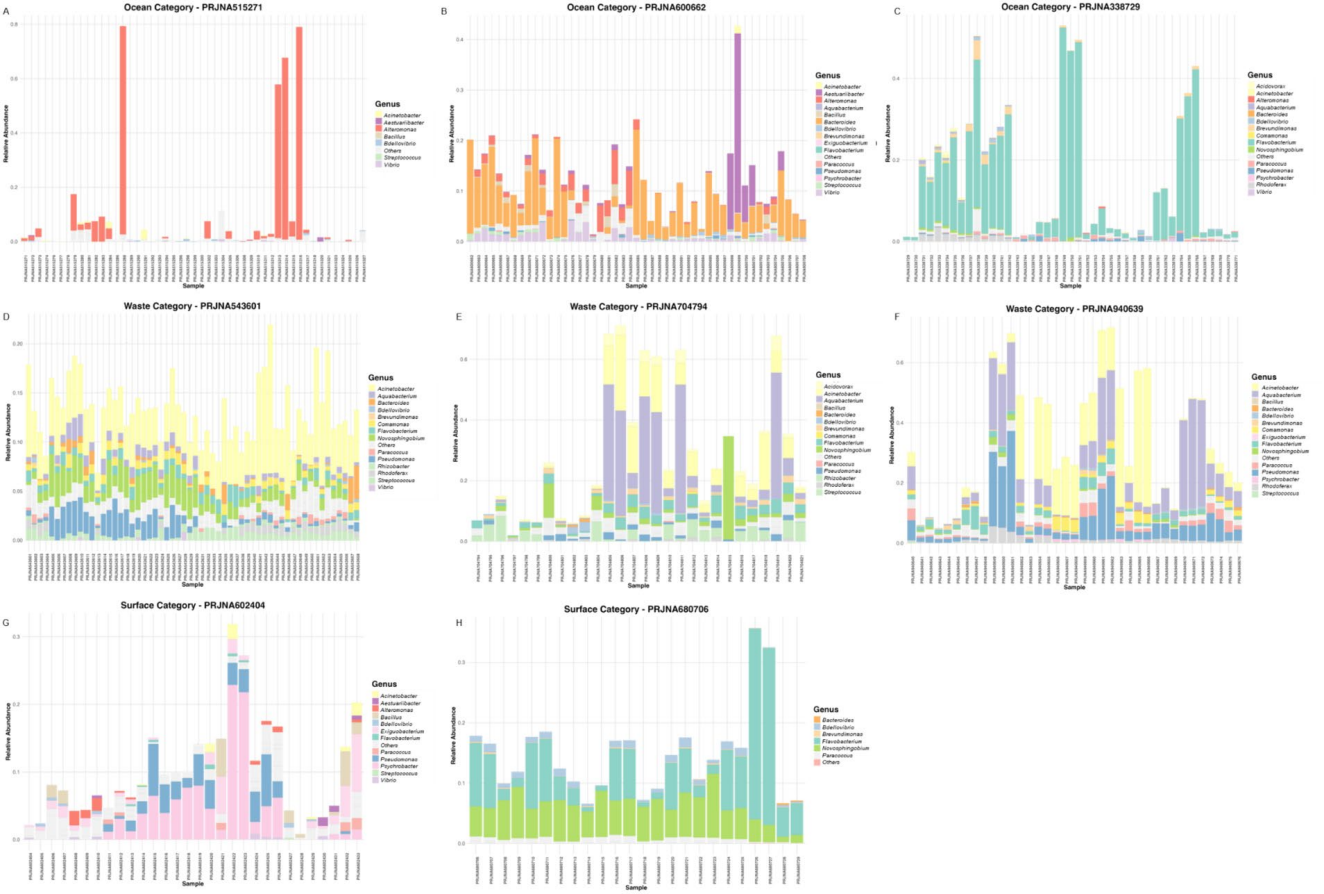
Relative abundance of potential PDB genera, expressed as percentage values (e.g., 0.2 = 20%), across plastic biofilm samples from ocean **(A–C)**, wastewater **(D–F)**, and surface water **(G–H)** environments. For an interactive version of the figure with improved font visibility, click on the dataset links: PRJNA515271, PRJNA600662, PRJNA338729, PRJNA543601, PRJNA940639, PRJNA704794, PRJNA602404, and PRJNA680706. PRJNA722376 was excluded from the plot due to absence of potential PDB genera.

The relative abundance of potential PDB genera exhibited distinct patterns among ocean, wastewater, and surface water environments (Figure 3). In ocean samples, the composition of potential PDBs varied considerably between different studies (or PRJNA datasets), with no single plastic-degrading genus dominating all samples. For example, some datasets showed a higher prevalence of *Alteromonas*, while others were dominated by *Flavobacterium or Bacteroides* (Figures 3A–C). Despite these genus-level variations of potential PDB, the dominant phyla within the overall microbial communities of ocean plastisphere samples were largely consistent across studies, primarily comprising Bacteroidota and Proteobacteria (Supplementary Figure S3). This variability suggests that the plastic-degrading capacity of marine biofilms is influenced by site-specific factors such as nutrient availability, polymer type, and environmental conditions (e.g., salinity and temperature).

In wastewater samples, potential PDB profiles were more consistent with *Pseudomonas*, *Acinetobacter*, and *Aquabacterium* frequently dominating the communities (Figures 3D–F). However, the dominant phyla within the overall microbial communities of wastewater plastisphere samples were diverse among studies (Supplementary Figure S4). Compared to ocean samples, the wastewater plastisphere exhibited greater evenness and richness, suggesting the co-existence of multiple plastic-degrading taxa. Notably, the relative abundance of potential PDB genera was generally higher (10.24 ± 6.61%) in wastewater compared to ocean (3.51 ± 5.36%) and surface water (3.97 ± 5.61%) samples (*p* < 0.001).

Surface water environments showed a broad diversity of plastisphere microbial phyla, with Bacteroidota, Proteobacteria, and Cyanobacteriota being the most prominent groups (Supplementary Figure S5). A diverse range of plastic-degrading genera was also observed (Figures 3G–H), with *Psychrobacter*, *Novosphingobium*, and *Bdellovibrio* as dominant genera. While a diverse community was detected, only genera successfully classified at the taxonomic level are presented here, reflecting the limitations of current taxonomy assignment approaches, particularly for surface water samples. The “Others” category encompasses a variety of low-abundance potential PDB genera, representing the broader microbial diversity. Furthermore, the presence of “Others” (Figure 3) and “Uncultured” (Supplementary Figures S2–S4) categories across all environments underscores a significant knowledge gap that warrants further investigation using advanced cultivation and genome-resolved techniques.

### 3.3 Interactions between potential PDB and NDB

**Figure 4:**
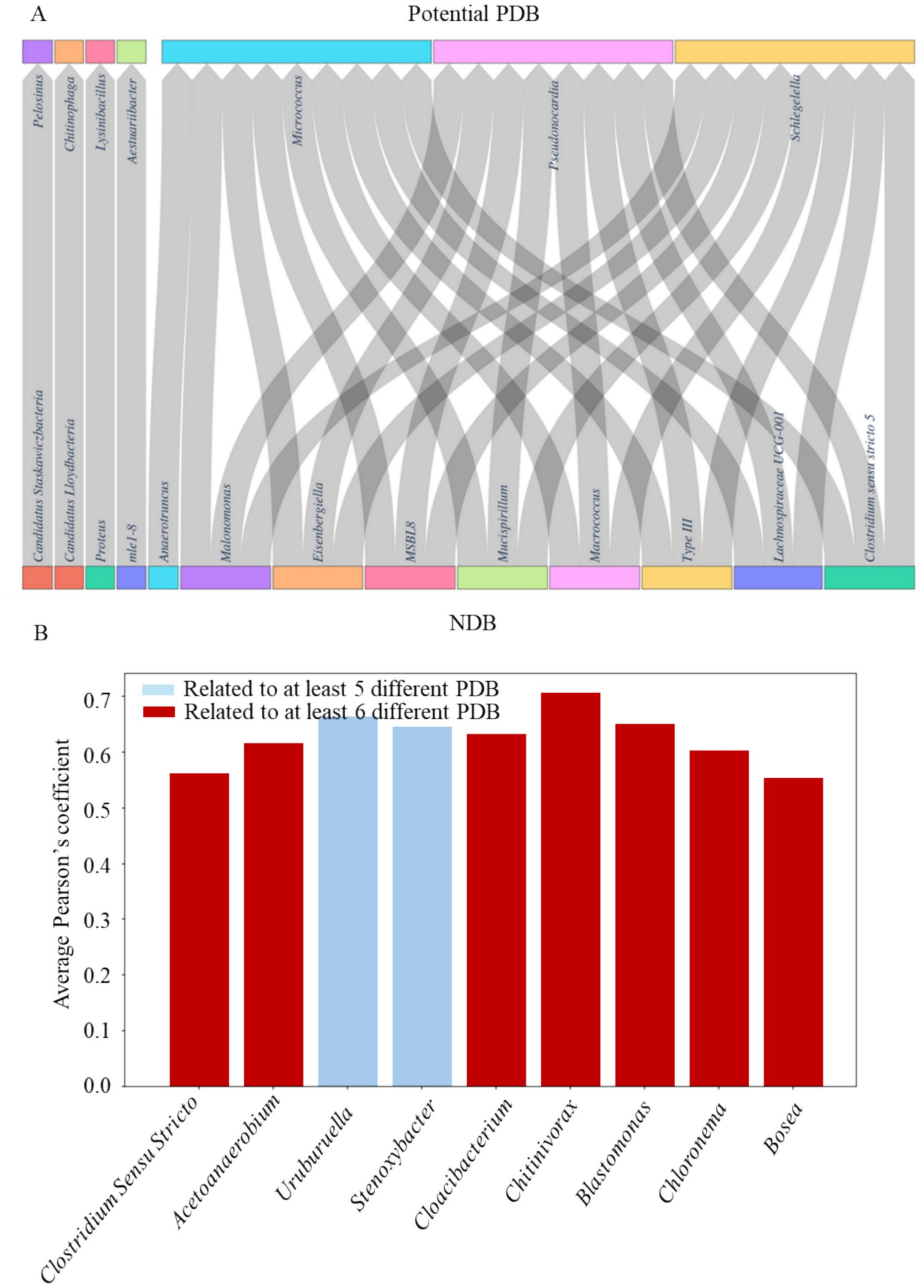
**(A)** NDB exhibiting Pearson’s correlation coefficient greater than 0.60 with potential PDB. **(B)** NDB show frequent interactions with more than five potential PDB with average Pearson’s correlation coefficient greater than 0.5.

Pearson’s correlation analysis identified several NDB genera exhibiting strong positive linear correlations with potential PDB (Pearson’s coefficient > 0.60), highlighting genera such as *Micrococcus*, *Pseudonocardia*, and *Schlegelella* (Figure 4A). These potential PDB genera were positively associated with several Firmicutes members (e.g., *Eisenbergiella, Macrococcus*, *type_III*, *Lachnospiraceae_UCG-001*, *Clostridium_sensu_stricto_5*), Cloacimonadota (*MSBL8*) and Deferribacterota (*Mucispirillum*). *Clostridium_sensu_stricto_5*, *Eisenbergiella*, *Anaerotruncus*, *MSBL8*, *Lachnospiraceae_UCG-001*, and *type_III* are associated with anaerobic or low-oxygen environments. In parallel, genera such as *Micrococcus*, *Chitinophaga*, *Clostridium_sensu_stricto_5*, and *Eisenbergiella* are linked to the degradation of complex organic matter, including proteins, carbohydrates, and polysaccharides (Lu et al., 2023; Minor et al., 2024; Zaplana et al., 2024; Kim et al., 2025). Many of these genera, including *mle1-8* and *MSBL8*, remain poorly characterized, highlighting the need for further investigation.

Several NDB genera demonstrated broad positive associations with potential PDB, suggesting potential ecological importance within plastisphere communities. *Urulurrella* and *Stenoxybacter* were associated with more than five potential PDBs (with average Pearson’s *r* > 0.65). Notably, *Clostridium_sensu_stricto*, *Acetoanaerobium*, *Cloacibacterium*, *Chitinivorax*, *Blastomonas*, *Chloronema*, and *Bosea* were each correlated with more than six distinct potential PDB genera, highlighting their centrality in the network.

**Figure 5:**
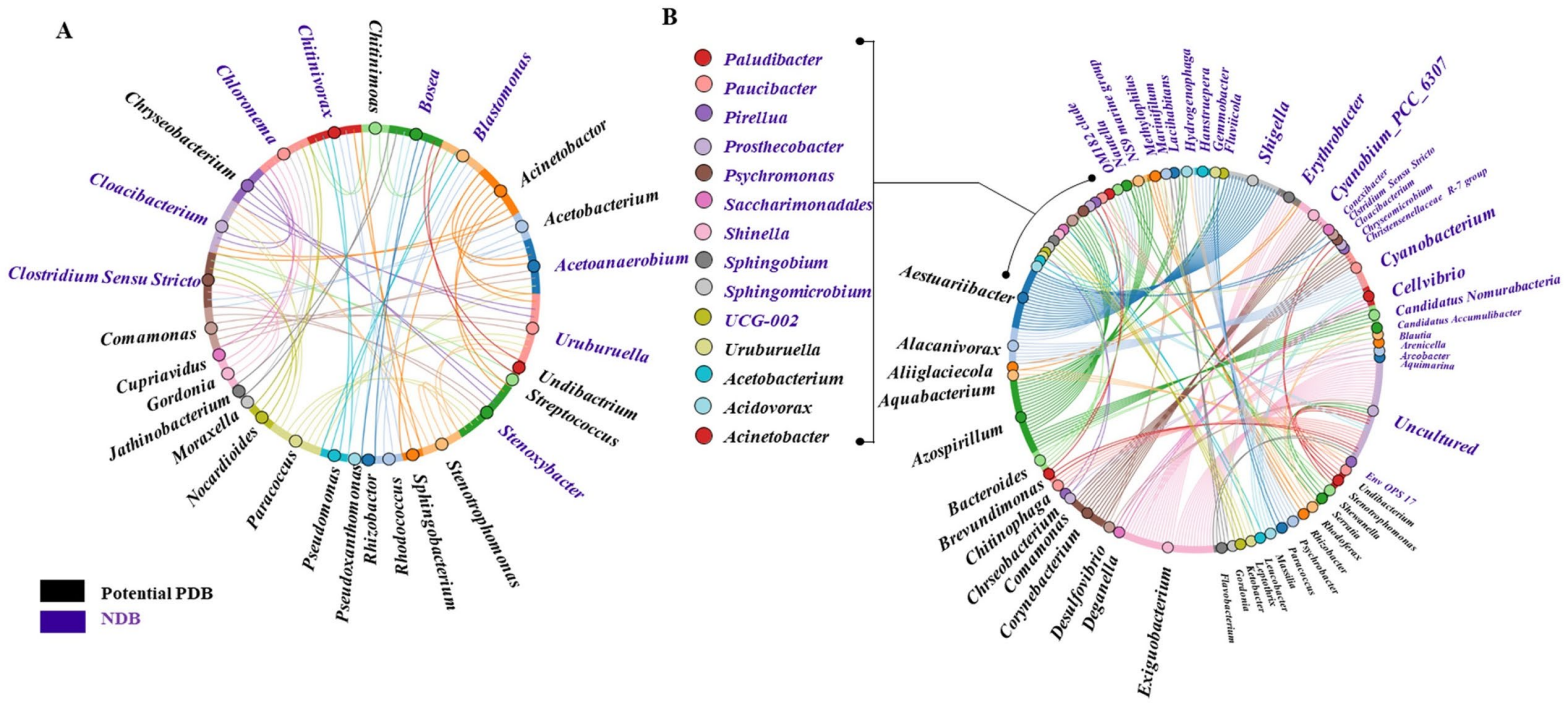
**(A)** Mutual linear interactions between potential PDB and NDB based on Pearson’s correlation analysis (*r* > 0.50). **(B)** Non-linear interactions between potential PDB and NDB identified through feature importance using random forest modeling. Font size is proportional to feature importance.

Mutual linear associations based on Pearson’s correlation coefficients (*r* > 0.50) revealed a dense network of co-occurrence patterns (Figure 5A). Notably, several NDB genera such as *Clostridium_sensu_stricto*, *Cloacibacterium*, *Bosea*, and *Blastomonas* exhibited extensive correlations with multiple potential PDB genera, suggesting potential cohabitation or supportive roles in plastisphere communities. This analysis was extended by identifying non-linear relationships through Random Forest modeling, where feature importance metrics highlight predictive interactions between NDB and potential PDB. Non-linear modeling approach uncovered additional associations not captured by linear correlation, including interactions involving less abundant or uncultured taxa (Figure 5B). For instance, *Aestuariibacter* (potential PDB) is associated with *Shigella* (NDB), while *Corynebacterium* (potential PDB) showed strong links to *Cyanobium_PCC_6307* (NDB) and *Cyanobacterium* (NDB). Additionally, *Exiguobacterium* (potential PDB) was connected to several uncultured NDB taxa, highlighting the potential ecological relevance of poorly characterized microorganisms in plastisphere networks.

In addition to their roles as potential plastic degraders, several genera identified in our analysis (e.g., *Pseudomonas*, *Acinetobacter*, *Aeromonas*, and *Bacteroides*) also include opportunistic or clinically relevant pathogens. Their enrichment on microplastic surfaces suggests that plastispheres may act as reservoirs not only for biodegradative potential but also for taxa of public health concern. While our genus-level resolution cannot distinguish pathogenic from non-pathogenic species, the co-occurrence of plastic-degrading capacity and pathogenic potential highlights the dual ecological roles of plastisphere microorganisms.

To minimize the influence of broad-scale taxonomic turnover between saline and non-saline systems, we conducted environment-specific analyses of PDB–NDB relationships (Supplementary Tables S1–S6), which highlighted recurring NDB genera potentially influencing PDB abundance within individual environments. Several NDB genera (e.g., *Hydrogenophaga*, *Prosthecobacter*, *Phaselicystis*) were detected across multiple environments but were associated with distinct PDB partners in each (Supplementary Table S7). Although the resulting co-occurrence and feature-importance patterns differed from those obtained using pooled data (Supplementary Table S7 compared with Figure 4), some consistent trends emerged. *Clostridium Sensu Stricto* appeared as a key NDB linked with six potential PDBs in both ocean and surface water environments, whereas *Acetoanaerobium, Uruburuella, Stenoxybacter, Cloacibacterium, Blastomonas, Chloronema*, and *Bosea* were predominantly associated with wastewater. In contrast, *Chitinivorax* was shared between ocean and wastewater environments. These shifts indicate that PDB–NDB relationships are strongly environment dependent, consistent with the separate clustering of ocean, surface-water, and wastewater networks.

### 3.4 Environment-dependent patterns in potential PDB and NDB communities

**Figure 6:**
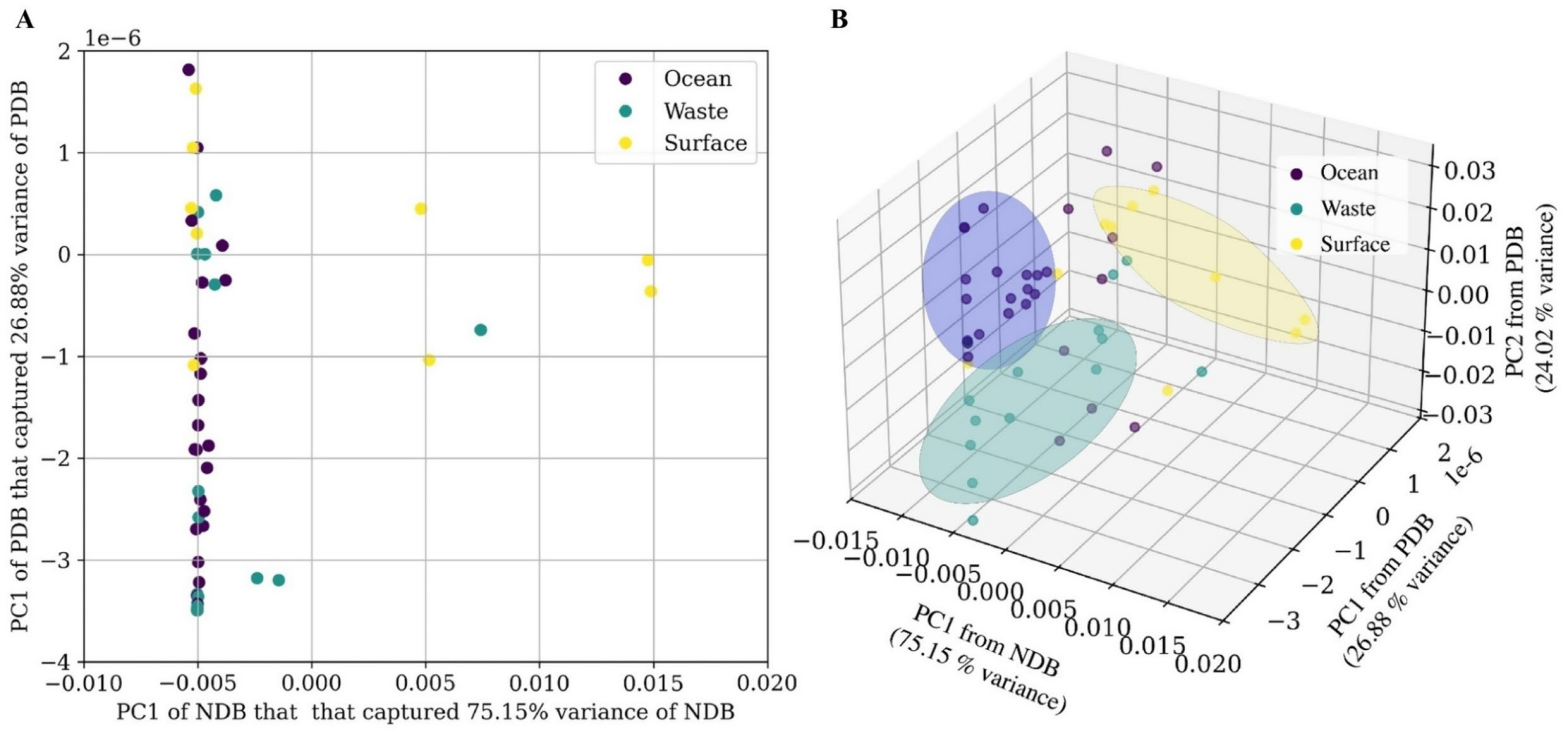
PCA of potential PDB and NDB abundance across different habitats. **(A)** 2D scatter plot showing the distribution of samples based on PC1 of NDB and PC1 of potential PDB. **(B)** 3D scatter plot incorporating an additional component (PC2 of PDB).

Normalized genus-level bacterial abundances from ocean, surface water, and wastewater samples were subjected to PCA for dimensionality reduction and then classified using the KNeighborsClassifier implementation in Scikit-learn. PC1 of NDB explained 75.15% of the total variance, while PC1 of potential PDB explained 26.88%, indicating that NDB features contributed more strongly to overall variation in the PC space. In the 2D PCA plot (Figure 6A), ocean samples clustered tightly, along the NDB axis, reflecting low intra-group variability and limited differentiation by NDB composition. In contrast, wastewater samples were more dispersed, especially along the potential PDB axis, suggesting greater community diversity and potential PDB–NDB co-occurrence. Surface water samples exhibited intermediate clustering patterns, influenced by both components. To improve separation and add more variability of potential PDB, a 3D PCA plot was generated incorporating PC2 of PDB (24.02% variance explained) alongside PC1 of NDB and potential PDB (Figure 6B). This enhanced visualization distinguished the three environments (PERMANOVA *p* < 0.001), with wastewater showing the greatest spread and surface water aligning more with the NDB axis, indicating a stronger role of NDB diversity in shaping those communities.

## 4 Discussion

### 4.1 Wastewater harbors the most diverse plastisphere microbial communities

Our results demonstrate that plastisphere microbial diversity significantly varies across different habitats (Figure 2). Plastisphere microbial communities in wastewater environments were the most diverse and even microbial communities (Figures 2A–D and Supplementary Figure S1) were consistent with previous studies (Besemer et al., 2012; Hannaford et al., 2023). This was due to the complex physicochemical composition, continuous influx of organic and inorganic nutrients, and high microbial seeding potential of wastewater, which supports a wide array of microbial taxa (Besemer et al., 2012; Hannaford et al., 2023). Wastewater contains a wide spectrum of carbon sources, nitrogen compounds, pharmaceuticals, heavy metals, and micropollutants, which collectively select for a broad range of microbial metabolisms. Prior research has highlighted that such environments promote microbial richness and functional potential, particularly in wastewater treatment plants where activated sludge harbors complex consortia dominated by *Proteobacteria*, *Bacteroidetes*, and *Actinobacteria* (Begmatov et al., 2022). However, because polymer identity/age and exposure history were heterogeneous across studies, and our habitat comparisons draw on three studies per habitat, we interpret these differences cautiously and avoid generalizing beyond this dataset.

Wastewater plastispheres not only reflect these background community characteristics but may further enrich specific taxa through surface attachment and biofilm formation on plastic substrates. Microplastics in wastewater have been shown to facilitate biofilm development that includes opportunistic pathogens, plastic-degrading microbes, and antibiotic resistance gene carriers, increasing ecological and public health relevance (Hong et al., 2023; Witsø et al., 2024). The relatively stable temperature and high particulate load in wastewater also enhance biofilm establishment and maintenance, leading to more even community structures. These conditions contrast with marine and surface water systems, which tend to be more oligotrophic (Sunagawa et al., 2015; Amaral-Zettler et al., 2020).

Freshwater environments are often characterized by seasonal fluctuations in nutrient levels, temperature, and flow rates, which can limit the stability and maturity of biofilms on plastic surfaces. In many lotic systems, hydrodynamic disturbances such as turbulence and sediment resuspension prevent long-term microbial colonization and biofilm succession, leading to communities dominated by a few fast-growing or surface-attached taxa (Jacquin et al., 2019). Freshwater habitats typically receive lower loads of organic and synthetic pollutants compared to wastewater, which reduces the chemical complexity and resource heterogeneity available to support a wide range of microbial niches (Amaral-Zettler et al., 2020; Henson et al., 2021; Browning and Moore, 2023). As a result, niche breadth and microbial interactions are constrained, yielding lower plastisphere diversity.

Compared to wastewater environments, the ocean plastisphere tends to harbor moderately diverse but compositionally stable microbial communities, shaped primarily by oligotrophic conditions and environmental filtering. Numerous studies have shown that marine environmental parameters, such as salinity, temperature, hydrodynamics, and seasonal variation, exert a stronger influence on plastisphere community assembly than plastic type or particle size (Du et al., 2022). These selective pressures favor the colonization of slow-growing, nutrient-efficient microorganisms, including *Oceanospirillales*, *Alteromonadales*, and *Alphaproteobacteria*—taxa that are consistently enriched on marine plastics across geographic regions (Wright et al., 2021).

Beta diversity analyses further supported the distinct clustering of plastisphere communities according to habitat type (Figure 2E, 6). Our results supported the conclusion that plastisphere microbial communities are influenced more by environmental conditions than by plastic substratum type. This aligns with (Wu et al., 2019), who showed that WWTPs host core microbiomes that are both taxonomically and functionally distinct from those in freshwater and marine systems. Plastisphere microbial communities in wastewater notably exhibited the greatest dispersion (Figures 2E, 6), highlighting that both NDB and potential PDB significantly contribute to community differentiation and variability. This aligns with previous studies demonstrating that wastewater environments are conducive to specialized plastic-degrading taxa alongside diverse non-degrading microorganisms, potentially supporting syntrophic relationships essential for efficient biodegradation processes (Yoshida et al., 2016; Danso et al., 2019). Variable influent composition, treatment stage heterogeneity, and elevated chemical complexity, driven by nutrients, pollutants, and other anthropogenic inputs, are known to foster highly diverse and adaptable microbial communities capable of colonizing and degrading complex contaminants such as plastics (Montazer et al., 2020; Gao and Sun, 2021).

In contrast, plastisphere microbial communities in ocean formed compact clusters predominantly along the NDB axis (Figures 2E, 6), indicating relatively uniform community structures characterized by low NDB variability. This suggests that ocean microbial communities might harbor consistent NDB compositions, possibly due to more stable and selective pressures compared to freshwater or wastewater ecosystems (Pinnell and Turner, 2019; Wright et al., 2020). Our results indicated that NDB diversity was the dominant factor shaping community differentiation across environments (Figure 6). Such findings suggest that the baseline microbial assemblage of each habitat has a stronger influence on plastisphere composition than the relatively small subset of taxa directly involved in plastic degradation. This pattern aligns with previous studies reporting that plastisphere biofilms are typically dominated by environmental generalists, while PDBs form a functionally important but minor fraction of the overall community (Wright et al., 2021; Du et al., 2022).

In comparison, the plastisphere microbial communities in surface water environments exhibited intermediate clustering patterns that overlapped with both ocean and wastewater samples, suggesting that surface water plastispheres may act as a transitional or mixing habitat. Surface water plastispheres may receive microbial inputs from both anthropogenic (wastewater) and natural (ocean or upstream) sources, leading to community structures influenced by a combination of selective and stochastic processes. For instance, Tulloch et al. (2024) found distinct shifts in plastisphere microbial community composition along the gradient—from wastewater effluent, through surface water, to marine exposure. Importantly, surface water plastisphere communities exhibited intermediate compositions, partly overlapping with both marine and wastewater microbial taxa (Tulloch et al., 2024).

Previous studies have demonstrated that understanding the associations between environmental factors and microbial community composition is essential for designing targeted bioremediation strategies and advancing ecological studies of plastic biodegradation (Montazer et al., 2020; Gao and Sun, 2021). In our study, wastewater environments represent the most promising habitat for developing such strategies. Their high microbial diversity, broad functional potential, and co-exposure to complex pollutants create favorable conditions for identifying, enriching, and engineering plastic-degrading microbial consortia.

### 4.2 Potential PDB profiles across habitats and their potential ecological roles

Genus-level analysis of plastisphere microbial communities and potential PDB across the three habitats revealed distinct taxonomic signatures and habitat-specific ecological trends (Figure 3 and Supplementary Figures S3–S5). In ocean environments, the dominant phyla within the plastisphere microbial communities were primarily Bacteroidota and Proteobacteria (Supplementary Figure S3). Many studies reported that Proteobacteria and Bacteroidetes dominated biofilms on both virgin and aged plastic materials deployed in seawater (Wright et al., 2021). Potential PDB genera such as *Flavobacterium*, *Alteromonas*, and *Bacteroides* were particularly abundant (Figures 3A–C). These taxa have been previously documented for their ability to colonize and degrade plastic surfaces in marine conditions, likely due to their metabolic flexibility and biofilm-forming capabilities (Zettler et al., 2013; Delacuvellerie et al., 2019). *Flavobacterium* spp. have been linked to the degradation of a variety of synthetic polymers like LDPE and polyurethane (Montazer et al., 2019). Based on PlasticDB, only one species of *Bacteroides* (Dwyer and Tiedje, 1986) has been reported to be associated with polyethylene glycol degradation, as determined by gas chromatography analysis. While members of the genus *Bacteroides* are typically anaerobic and gut-associated, their presence here may reflect input from fecal or wastewater sources or colonization of anaerobic microenvironments on plastic surfaces. *Alteromonas* species are frequently observed as early colonizers in marine plastisphere biofilms, often representing over 50% of the microbial community during initial exposure phases (Bos et al., 2023). Several *Alteromonas* strains, including *Alteromonas oceani*, have been experimentally associated with the degradation of plastics such as LDPE, Poly(3-hydroxybutyrate-co-3-hydroxyhexanoate), and polyurethane, supported by microscopy, FTIR, and GCMS evidence (Kato et al., 2019; Gunawan et al., 2022; Bitalac et al., 2023).

In contrast, potential PDB in wastewater showed a more homogeneous microbial profile, dominated by *Pseudomonas*, *Acinetobacter*, and *Aquabacterium*, even though the dominant phyla in wastewater plastisphere microbial communities can vary across studies (Figures 3D–F and Supplementary Figure S4). This pattern is consistent with previous findings that identify *Pseudomonas* (Kim et al., 2017; Ali et al., 2023) and *Acinetobacter* (Pramila, 2012; Montazer et al., 2018; Park et al., 2023) as frequent and effective plastic degraders in wastewater and landfill (Zhang et al., 2012). *Pseudomonas* species are widely recognized for their plastic-degrading capabilities across various polymer types, including PU, PHB, PE, PLA, and PVC blends. Notably, *P. aeruginosa*, *P. mendocina*, *P. protegens*, and *P. pseudoalcaligenes* have been isolated from diverse environmental sources such as soil, sewage sludge, and plastic waste, with multiple reports confirming their presence in plastisphere communities. Their broad environmental distribution and versatile metabolic capacity make *Pseudomonas* a functionally important genus for plastic bioremediation. Several *Acinetobacter* species, most notably *A. guillouiae* (strain PL211) and *A. nosocomialis* (strain GNU001), have been isolated from plastic-associated environments and shown to degrade low-density polyethylene (Seong et al., 2023; Kim et al., 2024). In addition, broader surveys have identified *Acinetobacter* spp. as previously reported plastic degraders across a variety of polymers such as LDPE, PCL, PE, PES, PET, PHA, PLA, PS, and PU (Wallbank et al., 2025). These genera have been extensively reported for their ability to degrade various plastic polymers through enzymatic processes, such as lipase, esterase, and monooxygenase activity (Urbanek et al., 2018; Danso et al., 2019). *Aquabacterium* species are frequently detected in biofilms and activated sludge. Recent evidence suggests that *Aquabacterium* species possess plastic-degrading enzymes related to PETase (Ho et al., 2023).

Our surface water plastispheres harbor dominant phyla such as Proteobacteria, Bacteroidota, and Cyanobacteria (Supplementary Figure S5), consistent with previous studies indicating that surface water plastisphere communities support diverse microbial phyla, including many low-abundance or uncultured taxa (Chen et al., 2021). Notably, genera such as *Psychrobacter*, *Novosphingobium*, and *Bdellovibrio* (Figures 3G–H) have also been detected in other freshwater plastisphere studies (Wang et al., 2018; Ou et al., 2024), highlighting the ecological complexity of these communities. *Psychrobacter* spp., frequently isolated from marine environments, have been associated with the degradation of PE, PCL, and PU (Zhang et al., 2022; Rüthi et al., 2023). *Novosphingobium* species are commonly found in freshwater sediments, lakes, and wastewater biofilms, where they are known for metabolizing aromatic compounds. Although plastic degradation by this genus is not widely reported, one strain of *Novosphingobium* sp. has been shown capable of PS degradation (Liu et al., 2023) when isolated from wastewater. Additionally, *Bdellovibrio bacteriovorus* has been experimentally confirmed to degrade polyhydroxyalkanoate, suggesting a possible role in bioplastic degradation (Martínez et al., 2012). However, its involvement in the degradation of conventional petroleum-derived plastics remains poorly characterized. As a predatory bacterium, *Bdellovibrio* may instead influence plastisphere structure through microbial predation and biofilm modulation rather than direct plastic degradation. Genus-level analysis of potential PDB across habitats reveals that wastewater serves as a reservoir of known degraders, ocean communities represent early colonizers, and surface water plastispheres are taxonomically diverse but remain functionally understudied.

### 4.3 Co-occurrence of NDB and potential PDB highlights biodegradation interactions

In our dataset, Pearson’s correlation and Random Forest analyses revealed consistent positive associations between several potential PDB genera, such as *Micrococcus*, *Pseudonocardia*, and *Schlegelella*, and diverse NDB taxa, including members of *Firmicutes* (e.g., *Clostridium_sensu_stricto_5*, *Eisenbergiella*, *type_III*, *Lachnospiraceae_UCG-001*), *Cloacimonadota* (e.g., *MSBL8*), and *Deferribacterota* (e.g., *Mucispirillum*) (Figures 4A,B). Many of these NDB taxa in our data analysis are adapted to anaerobic or microaerophilic environments and perform fermentative metabolism, producing short-chain fatty acids like acetate, lactate, succinate, and butyrate. For instance, *Clostridium_sensu_stricto_5*, *Eisenbergiella*, *Anaerotruncus*, *Lachnospiraceae_UCG-001*, *type_III*, and *MSBL8* in our datasets, are known to inhabit anaerobic or microaerophilic environments and engage in fermentative metabolism, producing volatile fatty acids such as acetate, lactate, succinate, and butyrate (Gao et al., 2022). They co-occurred with multiple potential PDBs, indicating that fermentative taxa may support degraders through nutrient or electron donor exchange. *Micrococcus*, *Chitinophaga*, *Lachnospiraceae_UCG-001* and *Anaerotruncus* are also known for degrading complex organic substrates such as proteins, carbohydrates, and polysaccharides (Lawson et al., 2004; Talamantes et al., 2016; Amaretti et al., 2019). These associations suggest that NDBs play a supportive ecological role in plastisphere biofilms. They may not degrade plastic directly but instead contribute by creating favorable conditions for potential PDBs, such as establishing redox gradients, supplying nutrients and growth factors, or removing inhibitory metabolites. Such facilitative interactions likely enhance the overall functional potential of plastisphere communities for plastic biodegradation, especially in low-oxygen or nutrient-rich habitats like wastewater. For instance, NDBs *Uruburuella* and *Stenoxybacter* are strongly and broadly associated with several potential PDBs (Figure 4A), implying roles in redox regulation or acetate cycling. For *Uruburuella*, functional roles remain speculative due to a lack of ecological studies, but these consistent correlations point to its potential importance in biofilm formation or microbial structuring (Silipo et al., 2012). However, *Stenoxybacter* may support plastic-degrading consortia by redox regulation or acetate turnover within plastisphere biofilms (Wertz and Breznak, 2007).

Mutual linear associations based on Pearson’s correlation coefficients (*r* > 0.50) revealed a dense network of PDB–NDB co-occurrence patterns across environments (Figure 5A). Notably, several NDB genera such as *Clostridium_sensu_stricto*, *Cloacibacterium*, *Bosea*, and *Blastomonas* exhibited extensive correlations with multiple potential PDB genera, consistent with supportive roles in plastisphere biofilms through cross-feeding, nutrient recycling, and biofilm stabilization. These findings align with prior studies showing that microbial plastic-degrading consortia often involve metabolically complementary taxa (Jacquin et al., 2019; Amaral-Zettler et al., 2020), where non-degraders facilitate colonization or breakdown indirectly through cross-feeding, nutrient recycling, or biofilm stabilization. For example, *Blastomonas* has been found in plastic biofilms in industrial recycling water (Tourova et al., 2021) and is known to be a pioneer colonizer with strong biofilm-forming potential, which may facilitate niche establishment for plastic-degrading species.

Unlike linear correlations, non-linear associations reflect complex, often indirect ecological relationships, likely indicative of metabolic dependencies, competitive interactions, or ecological co-selection pressures (Wright et al., 2020). For instance, *Aestuariibacter* (PDB genus), a genus within the *Alteromonadaceae* family with reported potential for PHA degradation, was associated with *Shigella* (NDB genus), a genus not commonly discussed in plastic degradation but potentially involved in nutrient cycling or microbial interactions within biofilms (Yasmin et al., 2025). Likewise, *Corynebacterium* (PDB genus) showed strong links to *Cyanobium_PCC_6307* and *Cyanobacterium*, genera known to participate in photosynthetic carbon fixation and redox regulation, possibly shaping the microenvironment of the plastisphere. Photosynthetic taxa such as *Cyanobium_PCC_6307* and *Cyanobacterium* may indirectly facilitate plastic degradation by producing oxygen and reactive oxygen species that oxidize polymer surfaces, enhancing microbial attachment and enzyme accessibility. Their extracellular polymeric substances and dissolved organic carbon exudates further enrich the plastisphere with labile substrates that support heterotrophic degraders such as *Corynebacterium*. These heterotrophs can co-metabolize oxidized polymers or utilize cyanobacterial exudates, driving complementary degradation processes. Similarly, *Microcystis* (Cyanobacteria) was detected on marine PET bottles, suggesting their role in modulating plastisphere functions through photosynthetic activity and toxin production (Kesy et al., 2016).

Additionally, *Exiguobacterium* (PDB genus) was connected to several uncultured NDB taxa. This is notable given that *Exiguobacterium* spp. have demonstrated broad substrate utilization capabilities and have been implicated in PS and LDPE degradation (Chauhan et al., 2018; Khandare et al., 2021), highlighting the potential ecological relevance of their interactions with under-characterized community members. These results suggest that poorly studied NDB taxa may play indirect but critical roles in plastisphere stability and function, echoing findings from microbial consortia studies (Urbanek et al., 2018) where syntrophic relationships enhance degradation efficiency.

There is growing interest in harnessing microbial activity to address plastic pollution, with the goal of developing more efficient enzymes and microbial systems for recycling facilities, landfills, and polluted ecosystems. However, our understanding of microbial interactions within the plastisphere, particularly how these interactions drive degradation functions, remains limited. Edwards et al. (2022) demonstrated that engineered microbial consortia outperform single isolates in PET degradation due to enzyme complementarity and cross-feeding (Edwards et al., 2022). Salini et al. (2024) enriched sewage sludge-derived consortia that significantly degraded PLA, highlighting the functional synergy of co-occurring taxa (Salini et al., 2024). Similarly, bacterial-bacterial combinations such as *Pseudomonas aeruginosa* RBM21 and *Bacillus subtilis* RBM2 have shown broad-spectrum plastic degradation capabilities, suggesting that interspecies interactions can expand substrate utilization and enhance enzymatic efficiency (Salinas et al., 2025). These findings align with our observations of consistent co-occurrence patterns between potential PDBs and NDBs, suggesting that plastisphere communities are structured by complex ecological interactions. Together, these results support the idea that efficient plastic degradation in natural environments likely depends on diverse microbial consortia rather than isolated degraders (Danso et al., 2019; Gao and Sun, 2021).

### 4.4 AI tools for understanding the interaction between potential PDB and NDB

ML has increasingly been applied to analyze microbial communities associated with plastics, particularly using 16S rRNA and metagenomic datasets. These approaches have enabled the classification of plastisphere taxa, prediction of potential plastic-degrading organisms, and inference of microbial interactions through co-occurrence network models such as Random Forests. For instance, Wright et al. (2021) analyzed over 2,200 plastisphere 16S rRNA samples from marine, freshwater, other aquatic (e.g., brackish or aquaculture) and terrestrial environments and found that hydrocarbon-degrading taxa like *Oceanospirillales* and *Alteromonadales* were consistently enriched on plastics (Wright et al., 2021). Their Random Forest analysis showed that environmental factors had a more substantial influence on community structure than plastic type, aligning with our findings that habitat context and PDB-NDB interactions are key in shaping plastisphere composition. However, this study did not include wastewater samples. Another study, using global 16S rRNA and ecological data from soil, seawater, and freshwater environments, found that plastisphere communities are taxonomically distinct, more heterogeneous and deterministically structured than surrounding habitats, characterized by specialist-dominated and fragile networks, although no ML models were applied in the analysis (Li et al., 2024).

AI models, particularly ML algorithms, are well-suited for detecting complex, non-linear patterns within high-dimensional ecological and microbial datasets, patterns that traditional statistical tools may overlook. By comparing our data with the PlasticDB database, we identified the distribution patterns of potential PDBs across ocean, surface water and wastewater environments, and applied ML models to explore their co-occurrence patterns and interactions with NDBs. However, this analysis reflects only the potential for plastic degradation. Further investigation is needed to confirm the presence and functional activity of specific PDB species. Given the limitations of database annotation and taxonomic resolution, the abundance of PDBs may be overestimated.

Despite the promise of ML, several bottlenecks limit its interpretability and functional relevance in plastisphere studies. First, taxonomic resolution from 16S rRNA gene sequencing data is often limited to the genus level, which does not accurately reflect functional potential, especially for plastic degradation. As a result, ML models may overestimate degradation capacity based on the presence of genera that include both degraders and non-degraders. Second, most interaction inferences are correlative rather than mechanistic, meaning co-occurrence does not confirm ecological interactions such as cross-feeding or enzymatic synergy. Third, habitat-specific biases, such as the high abundance of potential PDB genera in wastewater, can skew ML predictions, leading to overrepresentation of environmental associations that may not generalize across ecosystems. A further limitation is also the lack of integrated multi-omics approaches; most AI-based studies rely on taxonomic composition without incorporating functional genomic, transcriptomic, or metabolomic data, restricting our ability to draw conclusions about biodegradation pathways or microbial metabolism. Finally, environmental metadata, such as nutrient concentrations, oxygen levels, plastic types, or concentrations, is difficult to integrate into AI analyses due to its absence or lack of clear descriptions in public repositories like NCBI, thereby hindering context-aware ecological interpretations. Our findings are based on taxonomic inference and co-occurrence patterns without experimental validation. Future studies integrating metagenomic, transcriptomic, and enzyme-level analyses will help confirm true plastic degradation potential and refine functional understanding. To advance AI-powered plastisphere research, future studies must focus on improving data resolution, experimental validation, and the integration of multi-omics and environmental parameters to capture the true functional dynamics of microbial interactions on plastic surfaces.

## 5 Conclusion

Over the past several decades, the discovery of plastic-degrading enzymes and organisms have been driven by efforts to understand microbially mediated plastic degradation in the environment and to discover biocatalysts for plastic processing. This study demonstrates that plastisphere microbial communities are strongly shaped by habitat context, with wastewater systems supporting the highest microbial diversity and functional potential. Our genus-level profiling of potential PDBs revealed distinct taxonomic patterns across ocean, surface water, and wastewater environments. Critically, co-occurrence network analyses identified strong and consistent associations between potential PDBs and NDBs, suggesting that successful plastic degradation in the environment may depend on synergistic microbial interactions rather than isolated degraders alone. These ecological interactions may involve redox balancing, metabolite exchange, or structural biofilm support, enhancing the overall capacity for plastic degradation. ML approaches, particularly Random Forest models, enabled the identification of complex and non-linear relationships within microbial networks, offering valuable insights into plastisphere dynamics. However, current AI applications face challenges due to limited taxonomic resolution, database annotation gaps, and underutilization of multi-omics and environmental metadata. Overall, our genus-level, hypothesis-generating analysis nominates candidate PDB–NDB partnerships, highlighting the need for future polymer-resolved studies that integrate functional validation, metagenomics, transcriptomics, and detailed environmental data to advance toward predictive and actionable biodegradation strategies. Our results contribute to the growing body of evidence emphasizing that microbial consortia, not single species, will be essential for effective bioremediation of plastic pollution across diverse ecosystems.

## Data Availability

The data presented in this study are deposited in public repositories by previous studies. The 16S rRNA sequencing data are available from the NCBI Sequence Read Archive (SRA), and plastic-degrading microorganism data were obtained from PlasticDB. All the adopted datasets are listed in the Table 1 with their respective PRJNA accession numbers. The analysis scripts are available at https://github.com/skvarjun/ML_PDmicrobes.

## Footnotes

1
https://plasticdb.org/
