# Supplementary material for: Machine learning reveals microbial interactions driving plastic degradation across plastisphere environments
Source: Front Microbiol. 2026 Jan 23;16:1691658. doi: 10.3389/fmicb.2025.1691658 (PMC12876002; doi:10.3389/fmicb.2025.1691658)
Supplement: Supplementary file 1 [file Supplementary_file_1.docx]

Supporting information

Title:

Machine Learning Reveals Microbial Interactions Driving Plastic Degradation Across Plastisphere Environments

Authors: Akib AI Mahir^1,2┴^, Arjun Sathyan Kulathuvayal^3┴^, Yunjian Lei^4^, Qijun Zhang^5^, Luguang Wang^6^, Yanqing Su^3^, Liyuan Hou^1,2,6*^

^1^ Utah Water Research Laboratory, 1600 Canyon Road, Logan, UT 84321, USA

^2^ Department of Civil and Environmental Engineering, Utah State University, Logan, UT 84322, USA

^3^ School of Aerospace and Mechanical Engineering, University of Oklahoma, Norman, OK, 73019, USA

^4^ Computer Sciences Department, University of Wisconsin-Madison, Madison, WI 53706, USA

^5^ Department of Bacteriology, University of Wisconsin-Madison, Madison, WI 53706, USA

^6^ Department of Biological Engineering, Utah State University, Logan, UT 84322, USA

***Correspondence to:**

Dr. Liyuan Hou, liyuan.hou@usu.edu

Department of Civil and Environmental Engineering, Utah State University, Logan, UT 84322, USA

^┴^ Akib Al Mahir and Arjun Sathyan Kulathuvayal contributed equally to this work.


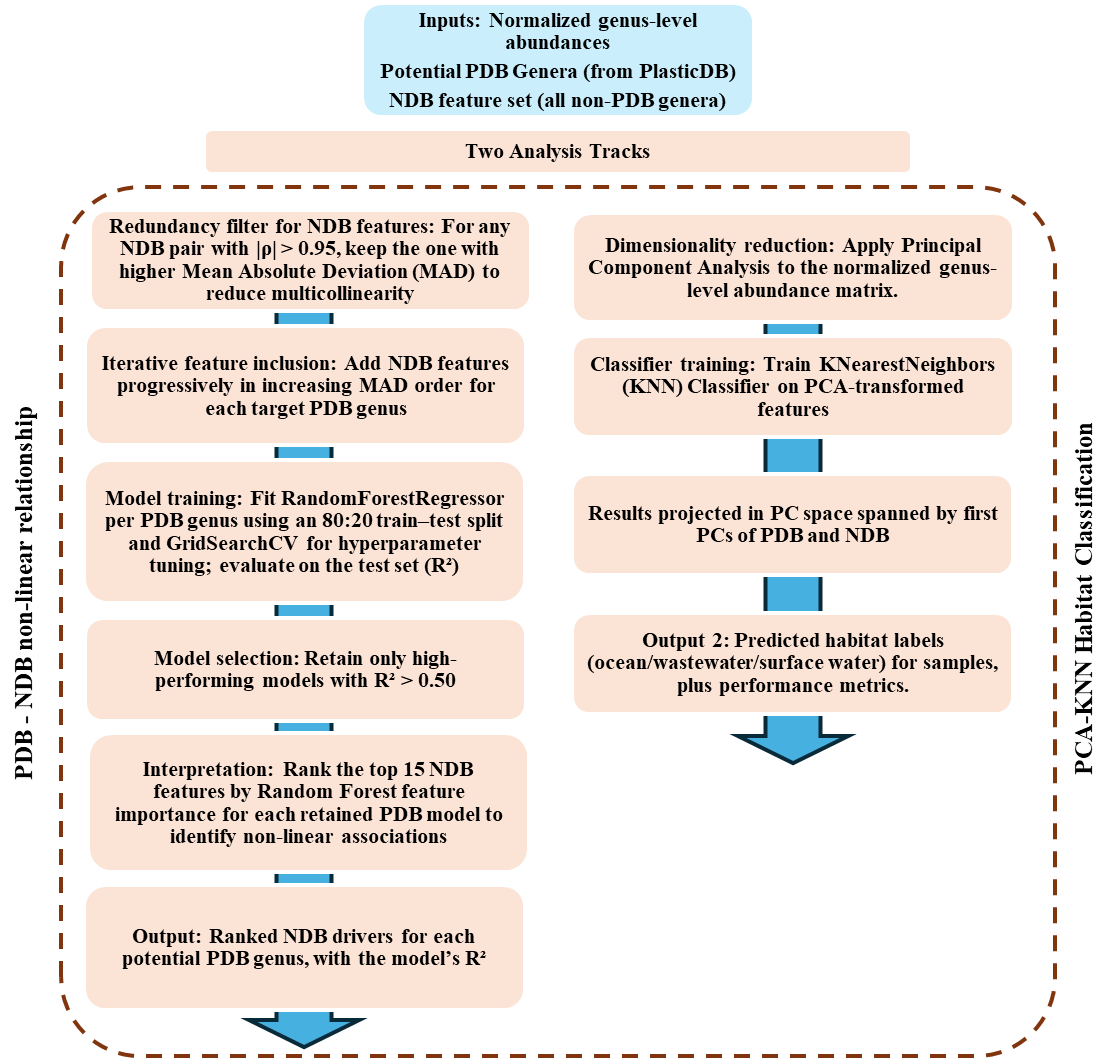


**Figure S1**. Workflow for machine learning analysis of potential plastic-degrading (PDB) and non-degrading (NDB) bacterial genera.
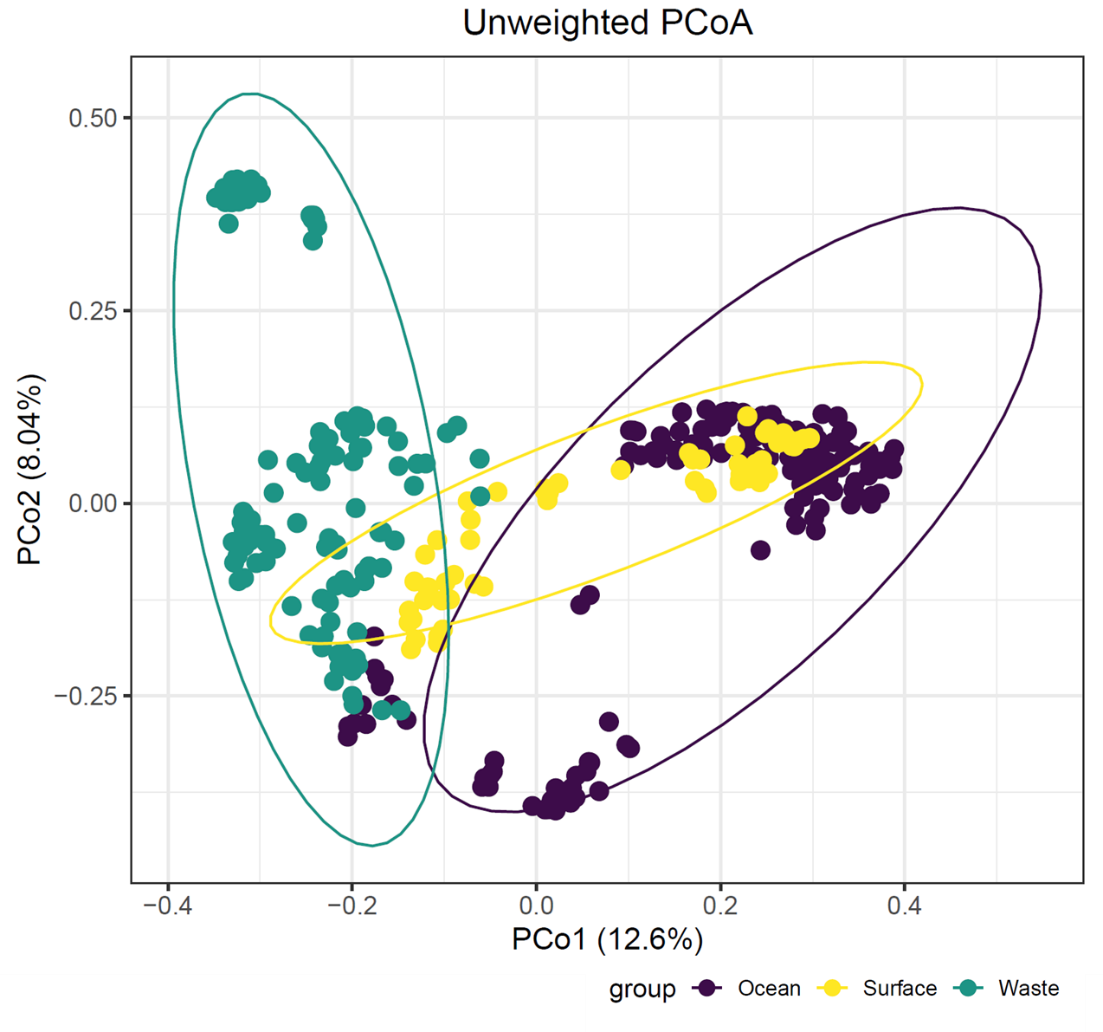


**Figure S2**. Principal Coordinates Analysis (PCoA) based on unweighted UniFrac distances showing beta diversity of plastisphere microbial communities across ocean, surface water, and wastewater environments.


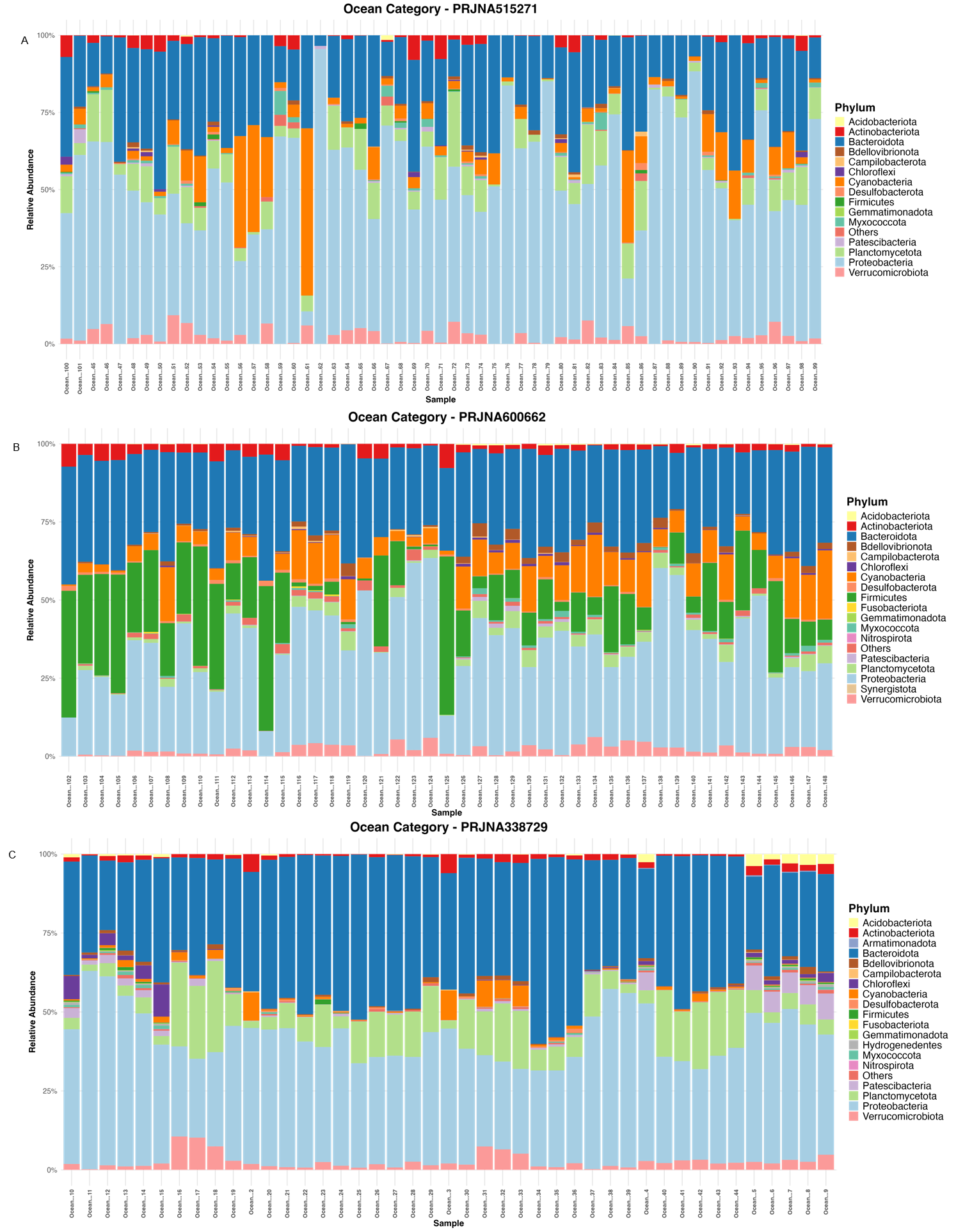


**Figure S3**. Relative abundance of bacterial phyla across plastisphere samples from ocean samples: [PRJNA515271](https://suddenlyappear0707.github.io/plastic-biofilm-plots-all-taxon/stacked_bar_plot_ocean_PRJNA515271.html) (A), [PRJNA600662](https://suddenlyappear0707.github.io/plastic-biofilm-plots-all-taxon/stacked_bar_plot_ocean_PRJNA600662.html) (B), and [PRJNA338729](https://suddenlyappear0707.github.io/plastic-biofilm-plots-all-taxon/stacked_bar_plot_ocean_PRJNA338729.html) (C). For an interactive version of the figure with improved font visibility, click on the dataset links: [PRJNA515271](https://suddenlyappear0707.github.io/plastic-biofilm-plots-all-taxon/stacked_bar_plot_ocean_PRJNA515271.html), [PRJNA600662](https://suddenlyappear0707.github.io/plastic-biofilm-plots-all-taxon/stacked_bar_plot_ocean_PRJNA600662.html), and [PRJNA338729](https://suddenlyappear0707.github.io/plastic-biofilm-plots-all-taxon/stacked_bar_plot_ocean_PRJNA338729.html).


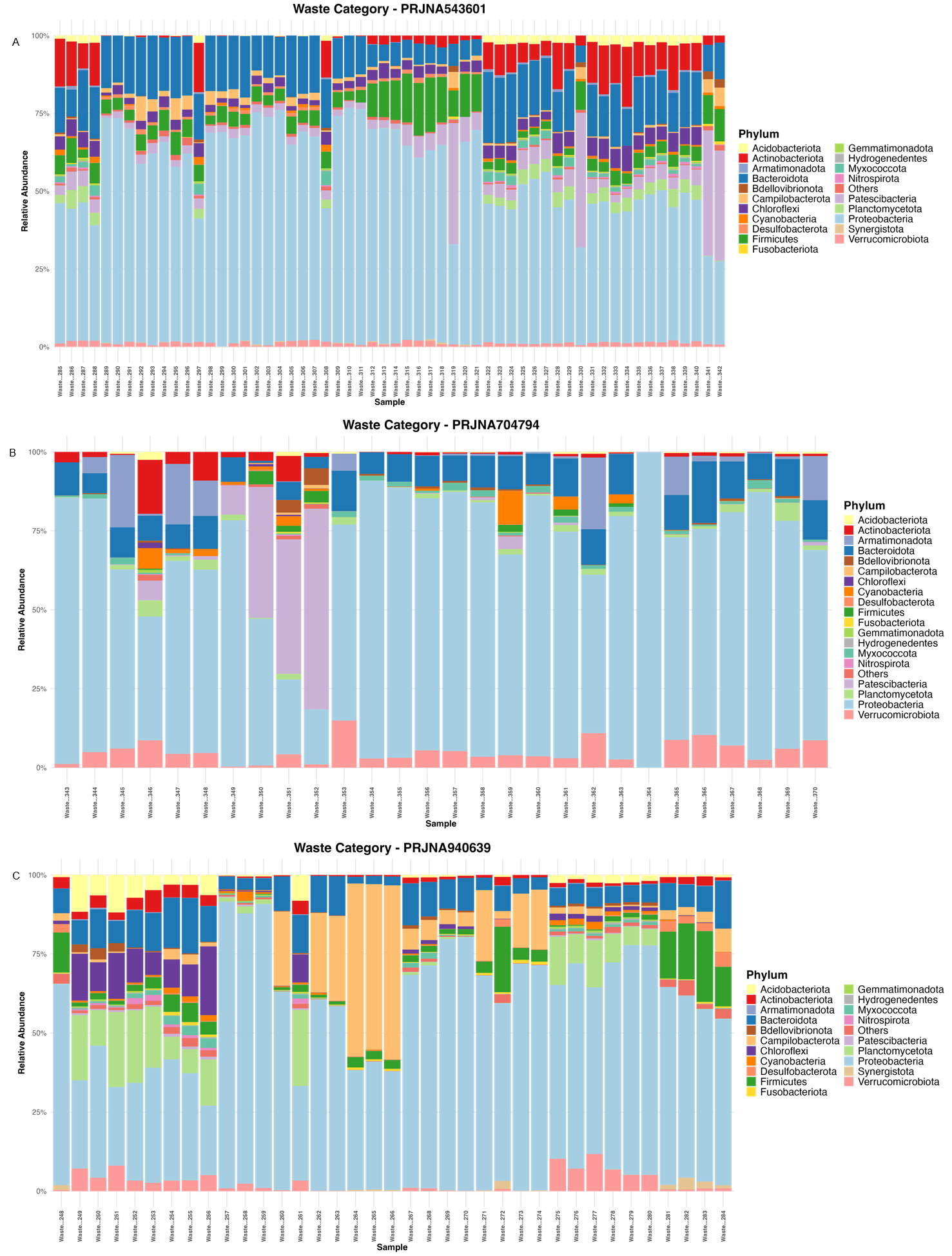

**Figure S4**. Relative abundance of bacterial phyla across plastisphere samples from wastewater samples: [PRJNA543601](https://suddenlyappear0707.github.io/plastic-biofilm-plots-all-taxon/stacked_bar_plot_waste_PRJNA543601.html) (A), [PRJNA704794](https://suddenlyappear0707.github.io/plastic-biofilm-plots-all-taxon/stacked_bar_plot_waste_PRJNA704794.html) (B), and [PRJNA940639](https://suddenlyappear0707.github.io/plastic-biofilm-plots-all-taxon/stacked_bar_plot_waste_PRJNA940639.html) (C). For an interactive version of the figure with improved font visibility, click on the dataset links: [PRJNA543601](https://suddenlyappear0707.github.io/plastic-biofilm-plots-all-taxon/stacked_bar_plot_waste_PRJNA543601.html), [PRJNA704794](https://suddenlyappear0707.github.io/plastic-biofilm-plots-all-taxon/stacked_bar_plot_waste_PRJNA704794.html), and [PRJNA940639](https://suddenlyappear0707.github.io/plastic-biofilm-plots-all-taxon/stacked_bar_plot_waste_PRJNA940639.html).

###
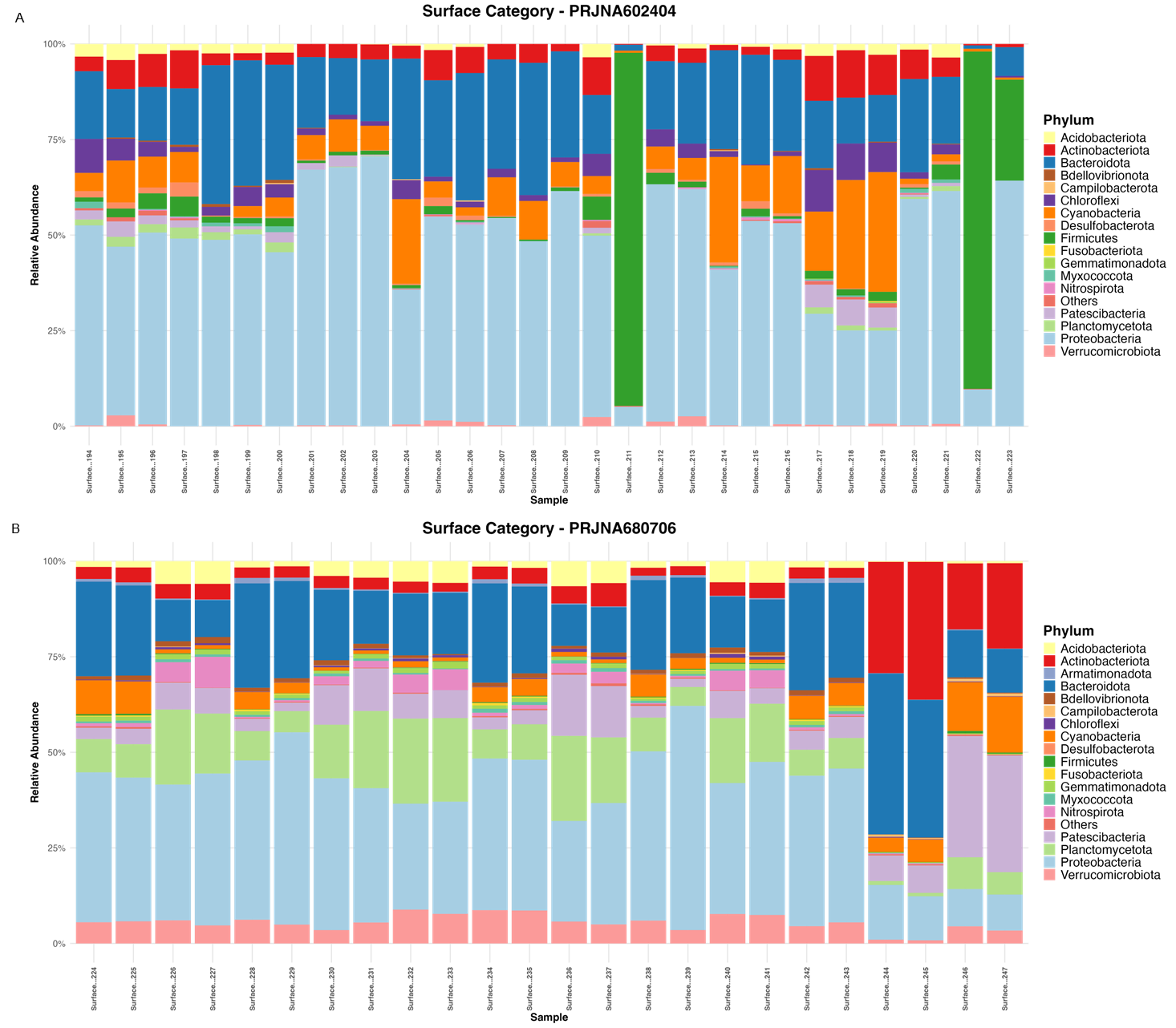


**Figure S5**. Relative abundance of bacterial phyla across plastisphere samples from surface samples: (A) [PRJNA602404](https://suddenlyappear0707.github.io/plastic-biofilm-plots-all-taxon/stacked_bar_plot_surface_PRJNA602404.html), (B) and [PRJNA680706](https://suddenlyappear0707.github.io/plastic-biofilm-plots-all-taxon/stacked_bar_plot_surface_PRJNA680706.html). PRJNA722376 was excluded due to insufficient data resolution at the phylum level for reliable analysis. For an interactive version of the figure with improved font visibility, click on the dataset links: [PRJNA602404](https://suddenlyappear0707.github.io/plastic-biofilm-plots-all-taxon/stacked_bar_plot_surface_PRJNA602404.html) and [PRJNA680706](https://suddenlyappear0707.github.io/plastic-biofilm-plots-all-taxon/stacked_bar_plot_surface_PRJNA680706.html).


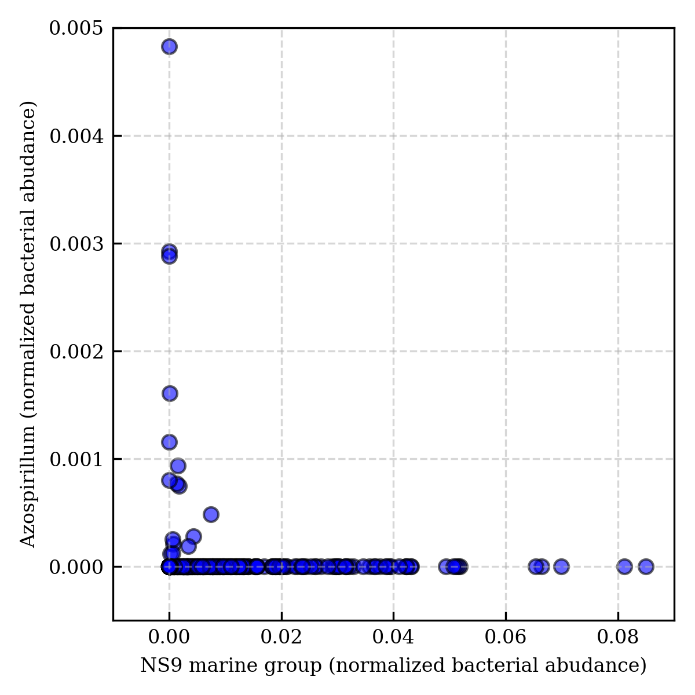


**Figure S6**. A non-linear association between *Azospirillum* (potential PDB) and the *NS9* marine group (NDB) based on normalized abundances detected by Random Forest analysis.

Table S1. Pearson correlation matrix between potential PDB and NDB genera in ocean samples.

Table S2. Random Forest–derived feature importance of NDB genera predicting PDB abundance in ocean samples.

Table S3. Pearson correlation matrix between potential PDB and NDB genera in surface water samples.

Table S4. Random Forest–derived feature importance of NDB genera predicting PDB abundance in surface water samples.

Table S5. Pearson correlation matrix between potential PDB and NDB genera in wastewater samples.

Table S6. Random Forest–derived feature importance of NDB genera predicting PDB abundance in wastewater samples.

Table S7. NDB associated with more than five distinct potential PDB for each environment.

See Supplementary Excel file (PDB_NDB_relationships_by_environment_sup.xlsx) for detailed results of Tables S1-S7.
